# Treatment and outcomes of patients with B-ALL relapse after CD19 CAR-T therapy

**DOI:** 10.1186/s12967-024-05362-w

**Published:** 2024-07-26

**Authors:** Yu Wang, Yu-juan Xue, Ying-xi Zuo, Yue-ping Jia, Ai-dong Lu, Hui-min Zeng, Le-ping Zhang

**Affiliations:** Department of Pediatrics, Peking University People’s Hospital, Peking University, No.11, Xizhimen South Street, Xicheng District, Beijing, 100044 China

Dear Editor,

Although CD19 chimeric antigen receptor-T lymphocytes (CAR-T) therapy has demonstrated remarkable therapeutic efficacy, the occurrence of B-cell acute lymphoblastic leukemia (B-ALL) relapse following CAR-T therapy remains a formidable challenge. In this study, we presented the clinical characteristics, post-CAR-T therapy, and evaluated outcomes of B-ALL patients who experienced relapse following CD19 CAR-T cell treatment at Peking University People’s Hospital.

Between June 2017 and March 2021, a total of 37 patients experienced relapse, with a median time from infusion to relapse of 10.3 months (range, 0.5–40.4 months). Detailed patient characteristics are provided in Table 1. Seventeen out of the 37 patients had previously undergone consolidative allo-hematopoietic stem cell transplantation following achievement of complete remission (CR) after a first round of CAR-T cell therapy. At the time of relapse, the CD19 immunophenotype on leukemia blasts was CD19 + in 17 cases, CD19-/dim in 10 cases, and CD19 status was unknown in 10 cases. A trend was observed towards a longer median time from CAR-T cell infusion to CD19 + relapse (10.6 months compared to 6.4 months for CD19- relapse). Bone marrow was the most common site of relapse after CAR-T therapy (*n* = 34), with extramedullary disease observed in 3 patients (8.1%), primarily involving the central nervous system (*n* = 2).

Among the 37 patients who relapsed, 18(48.6%) received subsequent salvage treatments, while 19 (51.4%) received supportive care or elected not to receive further therapy. Of the 18 patients who underwent post-CAR-T salvage treatments, 3(16.7%) received intensive multiagent chemotherapy, 10 (55.6%) were treated with either blinatumomab or inotuzumab, and 5 (27.8%) underwent reinfusion of CD19 CAR-T cells. Thirteen patients (72.2%) achieved CR, 4(22.2%) achieved partial remission (PR) and only 1 patient (5.6%) did not receive remission with salvage therapy. Following CR or PR, 10 patients underwent consolidative allo-HSCT (a first allo-HSCT, *n* = 3; a second allo-HSCT, *n* = 7). Two patients who underwent second allo-HSCT died due to transplantation-associated complications, 2 patient relapsed, and the other 6 patients remained alive in CR at the end of follow-up. Among the 6 patients who did not undergo transplant after attaining CR with immunotherapy (blinatumomab or inotuzumab or CD19 CAR-T), 4 experienced relapse and succumbed to progressive disease, and 2 patient remained alive in CR. The other two patients who did not bridge into transplantation after chemotherapy are currently alive, one of whom had central nervous system leukemia (CNSL) and the other relapsed three years after CAR-T treatment (Fig. 1).

The median time from relapse to last follow-up was 6.0 months (range, 1.0–74.7 months). Ten patients were still alive, while 27 died (*n* = 24, relapse; *n* = 3, treatment-related complications). The estimated 6-month and 12-month overall survival (OS) rates were (38 ± 8)% and (35 ± 8)% respectively. Different disease statuses prior to infusion have no effect on prognosis (12-month OS: MRD re-emergence^*^ vs. R/R B-ALL^#^, 27 ± 11% vs. 41 ± 10%, *P* = 0.746, * and # see Table 1). However, treated patients showed a significantly higher estimated 12-month OS rate compared to untreated patients [(72 ± 11)% vs. 0, *P* < 0.001]. Patients who underwent allo-HSCT after relapse exhibited better 12-month OS rates (80%±13%) compared to those who chose other treatments (12-months OS: 63%±17%) or received supportive care only (12-months OS: 0), *P* < 0.001.

CAR-T therapy has been proven to be the most effective strategy for R/R B-ALL patients. However, patients experiencing disease relapse after CAR-T therapy have a poor prognosis, and there is no standard treatment option post-CAR-T failure. Li W.J. et al. [1] explored the efficacy and safety of co-administration of CD22- and CD19-targeted CAR-T cells in 5 patients with B-ALL who relapsed after the first round CAR-T with the 12-month OS rate of 100%. Wudhikarn K. et al. [2] reported a 1-year OS of 35% in 38 adult patients with R/R B-ALL who experienced disease progression after CAR-T cell treatment, which was similar to the results observed in our study. Schultz L.M. et al. [3] conducted a retrospective multi-institutional study involving 57 patients who relapsed after CAR-T treatment, achieving a 1-year OS of 52%, surpassing the outcome observed in our center. This variance could be attributed to the fact that 88% of patients in their center received salvage therapy after relapse, while less than half of the relapsed patients in our center opted for subsequent salvage therapies. Consistent with Schultz L.M. et al. [3], immunotherapy remained the most common salvage treatment option in our study. Among these patients, ten were successfully bridged to HSCT after remission, 2 relapsed and 6 patients maintained MRD-negative CR at the end of follow-up. Conversely, among the 8 patients who did not undergo HSCT, 4 relapsed. However, Schultz L.M. et al. [3] did not observe a survival advantage in this subset of patients who underwent transplantation, as 37% of transplant recipients died and the specific causes of death were not specified.

In summary, CAR-T reinfusion can help patients achieving remission again, but the recurrence rate of immunotherapy alone is still high. The prognosis of the transplant group after remission appeared to be superior to that of the non-transplant group in our institution. Many attempts should be made to improve the duration of remission and decrease relapse rate following CAR-T cell treatment for B-ALL, including improving CAR-T products and/or combination treatments, and consolidative allo-HSCT.

**Fig. 1:**
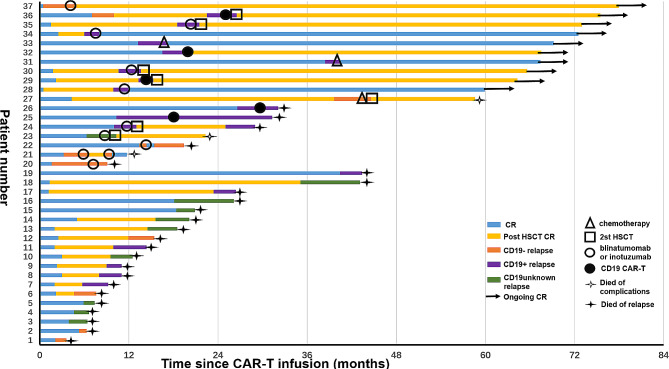
Treatment and outcomes in patients with B-ALL relapse after CD19 CAR-T therapy

**Table 1:** Clinical features of patients (*n* = 37)

	Patient	
	No	%
**Sex**		
Male	25	67.6
Female	12	32.4
**Age, y**		
Range	2–20	
Median	9	
**Fusion genes**		
BCR::ABL	2	5.4
ETV6::RUNX1	2	5.4
KMT2Ar	1	2.7
TCF3::PBX1	4	10.8
**EMD**		
Yes	3	8.1
No	34	91.9
**Disease status**		
MRD re-emergence^*^	15	40.5
R/R B-ALL^#^	22	59.5
**CAR-T origins**		
Patients	32	86.5
Donor	5	13.5
**Pretreatment MRD**		
MRD < 0.1%	12	32.4
MRD ≥ 0.1%	25	67.6
**Response to CAR-T at 30 days**		
MRD < 0.1%	35	94.6
MRD ≥ 0.1%	2	5.4

Abbreviations: MRD, minimal residual disease; R/R B-ALL, refractory/relapse B-cell acute lymphoblastic leukemia; CAR-T, Chimeric antigen receptor-T lymphocytes; EMD, extramedullary disease

* MRD re-emergence was defined as having at least two consecutive detectable MRD levels (sensitivity 1:10,000), despite achieving CR based on morphological assessment

# R/R B-ALL: Refractory ALL was defined as patients who failed to achieve CR after induction therapy. Relapsed ALL was defined as the reappearance of leukemia cells in peripheral blood or bone marrow (> 5%) or any extramedullary site after achieving CR

## Data Availability

The datasets are available from the corresponding author upon reasonable request.

## Abbreviations

R/R B-ALL: Refractory/relapsed B-cell acute lymphoblastic leukemia
Allo-HSCT: Allogeneic hematopoietic stem cell transplantation
OS: Overall survival
CR: Complete remission
PR: Partial remission
MRD: Minimal residual disease
